# The moderating effect of the COVID-19 pandemic on the mental wellbeing of health care workers on sustainable employability: A scoping review

**DOI:** 10.3389/fpsyt.2022.1067228

**Published:** 2023-01-06

**Authors:** Anneloes van den Broek, Louise van Hoorn, Yvette Tooten, Lars de Vroege

**Affiliations:** ^1^Department of Anxiety and Depression, GGz Breburg, Tilburg, Netherlands; ^2^Treatment and Coaching, Surplus, Breda, Netherlands; ^3^Department Gastro-enterology, ETZ Hospital (Elisabeth-TweeSteden Ziekenhuis), Tilburg, Netherlands; ^4^Department of Tranzo, Tilburg School of Social and Behavioral Sciences, Tilburg University, Tilburg, Netherlands

**Keywords:** healthcare workers, COVID-19, mental health, sustainable employability, resilience

## Abstract

Sustainable employability (SE) amongst healthcare workers (HCW) is an important asset for healthcare institutions. However, SE is under strain due to high work pressure, a shortage of employees, and absenteeism amongst employees based on mental problems. These developments had already started before the COVID-19 pandemic. The aim of this review is to explore whether there is a moderating effect of the COVID-19 pandemic on the mental wellbeing of HCW in the context of SE. A double blinded systematic review was conducted for this article in accordance with preferred reporting items for Systematic Reviews and Meta-Analyses (PRISMA) guidelines. Eligible studies were subjected to quality evaluation and narrative synthesis. The analysis of the selected literature led to the understanding that mental problems amongst HCW were already abundantly present before the COVID-19 pandemic. Mental health problems have increased in prevalence, severity, and variation. In general, a negative relation between (mental) health and SE exists. Our findings show that mental health problems have heavily impacted the SE of HCW: absenteeism has increased and perspective on work has changed. It is time to prioritize the mental health of HCW to prevent acute care capacity from declining even further and ending up in a vicious circle.

## Introduction

In the Wuhan region of China, the outbreak of the Coronavirus started in December 2019. Initially, there were only a few cases in Europe and the United States. However, in March 2020, the World Health Organization declared the COVID-19 outbreak a pandemic and varying periods of lockdown and social distancing followed with falling infection rates and new lockdown periods. In May 2020 China became virus-free, but during this time many European countries struggled with overcrowded hospitals followed by a decrease of infections in the summer and an increase of infections in the autumn of 2020, which led to new periods of lockdown. In December 2020, the first COVID-19 vaccination was carried out in the United Kingdom, which marked the start of a global immunization program and also brought hope and perspective for recovery. Given the global diversity in pandemic waves, government measures, and capacity in hospitals, countries were affected in different ways and also in different periods. One similarity between these countries, however, is the fact that the pandemic applied pressure on the healthcare system and resulted in cumulative staff loss. The COVID-19 pandemic required a great deal from healthcare workers (HCW). The healthcare system was overburdened for a long time, which confronted HCW with a high risk of burnout and threatened mental health severely (1). This persistent pressure for HCW and the mental overload gained attention. However, after months of continuous pressure, the resilience of hospital staff in coping with these problems and preserving their employees (as their primary resource) was exhausted (2). Moreover, after a decrease in COVID-19 infections and the related number of hospital admissions, the 'delayed care' in hospital and mental healthcare increased. This led to renewed pressure in hospitals and other sectors.

The peak in the development of mental health complaints followed the peak in the workload, as professionals initially responded to increased workload by fleeing into survival mode. Immers (3) indicated that 81% of all intensive care unit staff and employees were at risk of dropping out within 4 months due to considerable stress or burnout because they did not know how to cope with it. Recently, a survey amongst 1,300 Dutch HCW showed that, due to the COVID-19 pandemic, 50% reported increased levels of stress. Furthermore, a considerable amount of HCW were considering resigning from their jobs (4). The impact of HCW resigning from their jobs is potentially huge. From this perspective, sustainable employability (SE) becomes a hot topic. From SE perspective, the HCW can create possibilities to improve SE throughout their working lives by using their own capabilities. This creates in the conditions necessary for them to realize they can make a valuable contribution through their work while also taking care of their own (mental) health. Brouwers et al. (5) defined SE in a vitality assessment operationalizing five dimensions: “there should be a mix of competence and motivation, as well as resilience, mental and physical health, and social support at the workplace” (p. 1). The combination of the above-mentioned mental health problems in HCW and this operationalization gave a plausible reason to invest in (mental) healthcare for HCW, which is important for retaining staff for the longer term. For healthcare institutions, sustainable employable employees are of great importance, to promote the continuity and quality of care. The COVID-19 pandemic demanded a lot from HCW and as a result this might put pressure on SE. The results of a recent study by Faramazi et al. (6), emphasized that COVID-19 led to a high and significant economic burden by reducing productivity due to absence from work.

The aim of this study was to investigate whether there is a moderating effect of the COVID-19 pandemic on HCW mental health in the context of SE. We explored the moderating effects on SE of HCW in relation to the effects of COVID-19 on mental health and we discussed what these insights mean with regard to the SE of HCW. Several aims were developed. First, we aimed to explore which mental health problems amongst HCW existed before the COVID-19 pandemic and which prevalence rates are described in the scientific literature. Secondly, we aimed to explore which mental health issues amongst HCW existed as a result of the COVID-19 pandemic and what prevalence rates are described in the scientific literature. Thirdly, we explored SE of HCW in healthcare according to the scientific literature and aimed to explore the relationship between mental health of HCW and SE in healthcare.

## Materials and methods

The present international systematic literature review was carried out following PRISMA guidelines (7) in December 2021. After an explanatory search in Google Scholar and the Cochrane Library, the literature search was carried out in PubMed. PubMed is an English database with biomedical and life sciences' journal literature. The extensive search yielded professional literature; furthermore, articles were added from the expertise network of the researchers that had not (yet) been added to the scientific search engines, because these had recently been accepted for publication. Publications in the Dutch language were added because they were not found in PubMed. Each publication added to the dataset as described above was entered into the process (described below).

### Search strategy

The search terms (Table 1) were constructed by keywords [selected from dictionaries and the MeSH (Medical Subject Headings)] included in PubMed to index synonyms. The operators “And”/”OR” were used.

**Table 1:** Search terms.

	**Search string**	**Filter(s) applied**
Q1.	“Health Personnel”[Mesh] OR healthcare-professional*[tiab] OR health-care-professional*[tiab] OR healthcare-worker*[tiab] OR health-care-worker*[tiab] OR health-personnel[tiab]) AND (“Mental Health”[Mesh] OR mental-health-problem*[tiab]	Meta-Analysis, Randomized Controlled Trial, Review, Systematic Review, Dutch, English, from 1000/1/1 to 2019/10/1
Q2.	“Health Personnel”[Mesh] OR healthcare-professional*[tiab] OR health-care-professional*[tiab] OR healthcare-worker*[tiab] OR health-care-worker*[tiab] OR health-personnel[tiab]) AND (“Mental Health”[Mesh] OR mental-health-problem*[tiab]) AND (“Prevalence”[Mesh] OR prevalence[tiab]	Meta-Analysis, Randomized Controlled Trial, Review, Systematic Review, Dutch, English, from 1000/1/1 to 2019/10/1
Q3.	“Health Personnel”[Mesh] OR healthcare-professional*[tiab] OR health-care-professional*[tiab] OR healthcare-worker*[tiab] OR health-care-worker*[tiab] OR health-personnel[tiab]) AND (“Mental Health”[Mesh] OR mental-health-problem*[tiab]	Meta-Analysis, Randomized Controlled Trial, Review, Systematic Review, Dutch, English
Q4.	“Health Personnel”[Mesh] OR healthcare-professional*[tiab] OR health-care-professional*[tiab] OR healthcare-worker*[tiab] OR health-care-worker*[tiab] OR health-personnel[tiab]) AND (“Mental Health”[Mesh] OR mental-health-problem*[tiab]) AND (“Prevalence”[Mesh] OR prevalence[tiab]	Meta-Analysis, Randomized Controlled Trial, Review, Systematic Review, Dutch, English
Q5.	“Health Personnel”[Mesh] OR healthcare-professional*[tiab] OR health-care-professional*[tiab] OR healthcare-worker*[tiab] OR health-care-worker*[tiab] OR health-personnel[tiab])AND Sustainable-employability[tiab]	Meta-Analysis, Randomized Controlled Trial, Review, Systematic Review, Dutch, English
Q6.	“Health Personnel”[Mesh] OR healthcare-professional*[tiab] OR health-care-professional*[tiab] OR healthcare-worker*[tiab] OR health-care-worker*[tiab] OR health-personnel[tiab] OR healthcare-staff[tiab] OR health-care-staff[tiab] OR (healthcare[tiab] AND employee[tiab]) OR (health-care[tiab] AND employee[tiab) AND Sustainable employability[tiab] OR sustaining employability[tiab] OR sustained employability[tiab] OR (“Occupational Health”[Mesh] AND “Employment”[Mesh]) OR occupational-health[tiab] OR employee-health[tiab]	Review, Systematic Review, Dutch, English, 2020–2022

Q1; mental health problems amongst HCW before the COVID-19 pandemic, Q2; prevalence of mental health problems amongst HCW before the COVID-19 pandemic, Q3; mental health issues amongst HCW as a result of the COVID-19 pandemic, Q4; prevalence of mental health issues amongst HCW as a results of the COVID-19 pandemic, Q5; sustainable employability of HCW in healthcare, Q6; the relationship between mental health of HCW and SE in healthcare.

The search terms were constructed to collect material to provide specific answers to the questions formulated in this study. The inclusion criteria allowed the authors to select publications in English from several periods. The period differed for the separate questions: “Which mental health problems amongst HCW existed before the COVID-19 pandemic and to what prevalence?” referred to the period of 2008-2019; “Which mental health issues amongst HCW were as a result of the COVID-19 pandemic and of what prevalence” referred to the period of 2021; and “What is the SE of HCW in healthcare according to the scientific literature and how is the relationship between the mental health of HCW and SE in healthcare described?” referred to the period of 2020-2021.

### Inclusion and exclusion criteria

Meta-analyses, systematic reviews, literature reviews, narrative reviews, internet surveys, and Randomized Controlled Trials (RCT) were included. The exclusion criteria eliminated references with a wrong population according to the research questions (if not specified for HCW and “all inhabitants” was mentioned, Asian and African populations were excluded because of a different approach in diagnostics related to the use of DSM-5), as well as wrong study design and wrong publication type.

### Screening and quality appraisal

Three authors (AvB, LvH, and YT) independently screened the titles and abstracts in a blinded, standardized manner to determine eligibility using a standardized dating extraction form after implementing the articles in Rayyan (which is an Intelligent Systematic Review Program: https://www.rayyan.ai/). Disagreements between the reviewers were resolved after discussing the arguments between the specific reviewers and a second reviewer (LdV). After this critical appraisal the full-text papers were systematically evaluated to search relevant items after distribution amongst the reviewers. The following data were filtered: date of survey, first author names, country, study sample, sample sizes, measurement tools, and results of the study. The essential characteristics of the studies are summarized in Tables 2A–C.

**Table 2A:** Overview of the characteristics of the studies answering Q1 and Q2.

**Authors**	**Study design**	**Sample size (studies, total sample size)**	**Measurement instruments**	**Key findings**
Blackwelder et al. (8)	Overview	*N =* 7.288	Maslach Burnout Inventory	Q1. Burnout, depression, suicide, and substance abuse Q2. Rates at high levels
Brooks et al. (9)	Literature review	70 articles	Not applicable	Q1. Q2. Conflicting Reports and Mixed Data on the Prevalence of Mental Health Problems Among Physicians Compared to Non-Health Professionals
Buck et al. (10)	Review	19 articles	Not applicable	Q1. Burnout, depression, anxiety, suicide incidence Q2. Overall prevalence rates of depression that are similar to the general population. Q2. The suicide incidence rate for physicians is higher than that in the general population.
Gray et al. (11)	Realist Review	55 studies	Not applicable	Q1. Burnout, stress, and depression Q2. High rates
Grow et al. (12)	Literature review	98 articles	Not applicable	Q1. Burnout Q2. Unacceptably high; almost half of all physicians reporting burnout
Kim et al. (13)	Randomized Controlled Trial	2 surveys, *n =* 1,599	20-item RCES-D screening scale	Q1. Depression Q2. Mixed findings
Mateen and Dorji (14)	Review	13 articles	Not applicable	Q1. Burnout Q2. About 25% or more of physicians and other health-care workers are considered “burnt out”
Mihailescu et al. (15)	Scoping review of literature	91 articles	Not applicable	Q1. Burn-out, depression and suicidal ideation, distress Q2.
O'Connor et al. (16)	Systematic review and meta-analysis of prevalence and determinants	33 studies, *n =* 9,409	Maslach Burnout Inventory	Q1. Burn-out per dimension: Emotional exhaustion, depersonalization, low levels of personal accomplishment Q2. 40% for emotional exhaustion; 22% for depersonalization; 19% for a low level of personal competence
Rössler (17)	Systematic review and meta-analysis	31 articles	Maslach Burnout Inventory	Q1. Burn-out, post-traumatic stress symptoms, distress, depression Q2. Burnout increased significantly; post-traumatic stress symptoms reported by most mental health professionals; urgency 25%; depression 44.6%
Stewart et al. (18)	Review conceptual models	7 models	Not applicable	Q1. physician wellbeing Q2.

Q1; mental health problems amongst HCW before the COVID-19 pandemic, Q2; prevalence of mental health problems amongst HCW before the COVID-19 pandemic, Q3; mental health issues amongst HCW as a result of the COVID-19 pandemic, Q4; prevalence of mental health issues amongst HCW as a results of the COVID-19 pandemic, Q5; sustainable employability of HCW in healthcare, Q6; the relationship between mental health of HCW and SE in healthcare.

**Table 2B:** Overview of the characteristics of the studies answering Q3 and Q4.

**Author**	**Study design**	**Sample size (studies, total sample size)**	**Key findings**
da Silva et al. (19)	Meta-Analysis systematic review	21 studies	Q3. Anxiety, being quickly overstimulated, insomnia, (mortal-)fear were mentioned, likely related to extremely high workloads and the lack of protective equipment Q4. high rates of psychological distress.
de Vroege et al. (4)	Internet Survey	*N* = 1,372	Q3. Distress, anxiety, depression Q4. A high level of distress (50%), anxiety (14%), and depression (30%) amongst employees in mental healthcare
Drudi et al. (20)	Literature review	90 studies	Q3. Anxiety, burnout, Q4. high levels of anxiety, as well as burnout
Dutta et al. (21)	Systematic review and meta-analysis	1,958 studies, of which 33 studies including *N =* 39,703	Q3. Depression, anxiety symptoms, poor sleep quality followed by insomnia and stress among HCWs Q4. A high prevalence of depressive complaints, anxiety, poor sleep quality resulting in insomnia, and stress were reported.
Hao et al. (22)	Systematic Review and Meta-Analysis	20 studies, *n =* 10,886	Q3. HCW have depression, anxiety, insomnia, post-traumatic stress symptoms, phobia, obsessive-compulsive symptoms, and somatization symptoms Q4. Relatively high prevalence rate
Li and Scherer (23)	Systematic review and meta-analysis	65 studies, *n =* 97,333	Q3. Moderate depression, anxiety, and PTSD Q4. A high prevalence
Marvaldi et al. (24)	Systematic review and meta-analysis	70 studies, *n =* 101,017	Q3. Anxiety; depression; acute stress; post-traumatic stress, sleep disorders Q4. 30 % of anxiety (24.2–37.05); 31.1 % of depression (25.7–36.8); 56.5 % of acute stress (30.6–80.5); 20,2% of post-traumatic stress (9.9-33.0); 44.0 % of sleep disorders (24.6–64.5)
Olay et al. (25)	Systematic Review and Meta-Analysis	57 studies	Q3. Depression, Q4. The pooled prevalence in HCW 24% (95% CI: 20–28%): differentiated for function: 43% for frontline professionals (95% CI: 28–59%), 25% for nurses (95% CI: 18–33%), and 24% for physicians (95% CI: 16–31%), and.
Raoofi et al. (26)	Systematic review plus meta-analysis.	46 studies, *n =* 61,551	Q3. Anxiety Q4.Prevalence among HCWs was 26.1% (95% CI 19% to 34.6%)
Santabárbara et al. (27)	Meta-Analysis, Systematic Review	71 studies	Q3. Anxiety, Q4. HCW experience significant levels of anxiety, especially those on the frontline and nurses.
Saragih et al. (28)	Systematic Review and Meta-Analysis	38 studies	Q3. PTSD was reported as psychological complaints which occurred most frequently, followed by anxiety, depression, and distress. Q4. The pooled prevalence of mental health complaints for PTSD 49%, distress 49%, anxiety 40%, and depression 37%.
Styra et al. (29)	Cross-sectional, multi-centered hospital online survey	*N =* 3,852	Q3. Moderate/severe scores for symptoms of PTSD; Anxiety, depression Q4. Amongst HCW complaints like PTSD (50.2%), depression (31.5%) and anxiety (24.6%), were reported
Wu et al. (30)	Systematic Review and Meta-Analysis	66 studies, *n =* 221,970	Q3. Depression, anxiety, distress, and insomnia Q4. Pooled prevalence was 31.4%, 31.9%, 41.1% and 37.9%, the prevalence of insomnia in respectively Physicians, nurses, and non-medical staff was higher (Q = 196.64, *p* < 0.01) than other populations
Yan et al. (31)	Systematic Review and Meta-Analysis	35 studies, *n =* 25,343	Q3. Ear-related symptoms, stress, anxiety symptoms,; insomnia,; posttraumatic stress disorder symptoms,; depressive symptoms, and somatic symptoms, Q4. Ear-related symptoms, 67; stress, 56; anxiety symptoms, 41%; insomnia, 41%; posttraumatic stress disorder symptoms, 38%; depressive symptoms, 27%; and somatic symptoms, 16%

Q1; mental health problems amongst HCW before the COVID-19 pandemic, Q2; prevalence of mental health problems amongst HCW before the COVID-19 pandemic, Q3; mental health issues amongst HCW as a result of the COVID-19 pandemic, Q4; prevalence of mental health issues amongst HCW as a results of the COVID-19 pandemic, Q5; sustainable employability of HCW in healthcare, Q6; the relationship between mental health of HCW and SE in healthcare.

**Table 2C:** Overview of the characteristics of the studies answering Q5 and Q6.

**Author**	**Study design**	**Sample size (studies, total sample size)**	**Measurement instruments**	**Key findings**
de Vroege et al. (4)	Survey	*N =* 1372		Q5.— Q6. 11.3% agreed and 9.0% agreed that they were taking more sick leave. 7.5% agreed and 12.4% agreed that they should take more leave. Between 7.8% (partly agree) and 8.8% (agree) say they are more absent during the pandemic. 7.5% of respondents said they would consider working less and 4.2% said they would consider stopping working in a nursing home
Moloney et al. (32)	Integrative review	20 studies		Q5. Sustainability and organizational effectiveness through healthy, high-performing and engaged employees Q6. Internationally, nursing is faced with unsustainable working conditions, high staff turnover and staff stress factors that hinder effective care and negatively impact health outcomes. The workload of nurses is often heavy and demanding, leaving them physically and mentally exhausted. Nurses are more prone to injuries and physical illness than the general public due to stress, burnout and psychological imbalances, and are more prone to mental illness than the general public. A severe shortage of nurses is expected, which will increase the demand for surplus nurses and increase staff shortages and the willingness to leave. COVID-19 has exacerbated these problems.
Roczniewska and Richter (33)	Multilevel analysis	*N =* 269	Job Demands-Resources model (JDR-model)	Q5. Sustainable employability (SE) is defined as the opportunity for employees to make valuable contributions through their current and future jobs, while maintaining their health and wellbeing. SE includes good physical and mental health and wellbeing at work (job satisfaction). Q6. Hypothesis 1. Vertical trust at the individual level is positively associated with SE, namely (a) job satisfaction, (b) health and (c) job performance. Hypothesis 2. Team-level teamwork is positively associated with SE, i.e. (a) job satisfaction, (b) health and (c) job performance. Hypothesis 3. Transformational leadership at group level is positively associated with sustainable employability, i.e., (a) job satisfaction, (b) health and (c) job performance.
Schouten et al. (34)				Q5. — Q6. In particular, physical (lifting, standing) and mental stress (violence and aggression by third parties) and the use of harmful substances are important factors that explain the decline in sustainable employability.
Smyth and Pit (35)	Qualitative study	*N =* 19	Work ability (WA) model	Q5. Sustainable employability means that employees can realize tangible opportunities in the form of a range of competencies during their working lives. They also enjoy conditions that allow them to make a valuable contribution through their current and future work, while maintaining health and wellbeing. This requires on the one hand a suitable work environment for them and on the other hand the attitude and motivation to take advantage of these opportunities. An individual's SE is defined as the long-term ability to obtain, create, and retain work by adapting to changing employment, economic, and personal circumstances at different stages of life. Q6. A balance between lifestyle and work leads to better physical health (first floor) and a better attitude to work.
Van Dorssen-Boog et al. (36)	Project	*N =* 109	JDR-model	Q5. Employees are sustainable if they are not only able and willing to meet the demands of their current job, but also remain competent and motivated to remain productive throughout their working lives. Q6. We expect a virtuous cycle to increase motivation, vitality, health, and productivity as employees and teams learn to use resources more effectively such as autonomy, social support, and their own and others' strengths. In addition, we expect fewer negative outcomes such as burnout, absenteeism, and staff turnover/dismissal. Effective self-leadership behavior goes hand in hand with positive and constructive thinking.

Q1; mental health problems amongst HCW before the COVID-19 pandemic, Q2; prevalence of mental health problems amongst HCW before the COVID-19 pandemic, Q3; mental health issues amongst HCW as a result of the COVID-19 pandemic, Q4; prevalence of mental health issues amongst HCW as a results of the COVID-19 pandemic, Q5; sustainable employability of HCW in healthcare, Q6; the relationship between mental health of HCW and SE in healthcare.

The first reviewer, LvH, (2nd AvdB and 3rd YT) carried out a critical appraisal of mental health problems and prevalence before COVID-19; the second reviewer AvdB (2nd LvdH and 3rd YT) did the same with regard to mental health problems and prevalence during COVID-19, and the third reviewer YT (2nd LvdH, 3rd AvdB, and 4th LdV) critically appraised mental health and the relationship with SE.

## Results

### Study selection

#### Mental health problems amongst HCW before the COVID-19 pandemic and their prevalence before COVID-19

**Figure 1:**
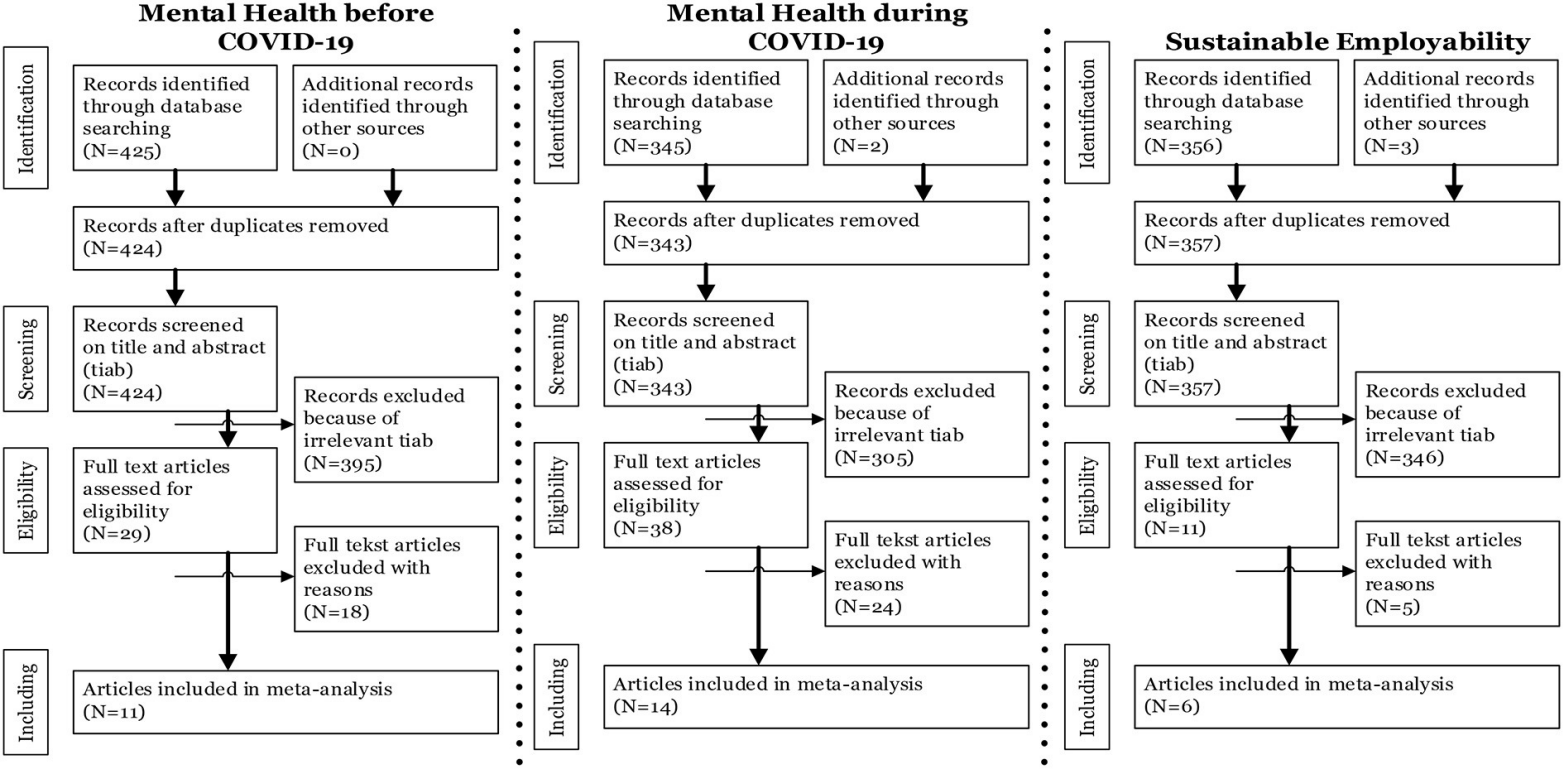
PRISMA flowchart of citations selection.

Figure 1 displays the PRISMA flowchart of the citations selection. Table 2A displays the total amount of research papers which were reviewed in order to explore what kind of mental health problems existed amongst HCW before the COVID-19 pandemic. This search yielded 425 peer-reviewed journal articles, including one duplicate study (which was deleted). Following the inclusion and exclusion criteria, first after screening titles and abstracts, 395 papers were excluded that were not relevant to the research question (e.g., studies related to the wrong population (not specified to HCW), studies related to a specific group of HCW, and/or studies related to a different angle [patient safety, costs, prevention, or interventions)]. After double blind screening abstracts for eligibility, another nineteen articles were excluded (irrelevant topics, like intervention studies or effect studies [Randomized Controlled Trials)]. A total of 12 English articles were reviewed. Ten of them described the mental health problems amongst HCW before the COVID-19 pandemic and one of them described the prevalence of mental health issues amongst HCW before the COVID-19 pandemic.

Five papers were defined describing a higher risk and prevalence of mental problems amongst HCW (8, 10, 12, 16, 17). One paper described mixed findings and suggested further research (9) and another paper described a similar level of complaints to that of the general population prevalence regarding depression amongst doctors (10). Mental health outcomes mostly mentioned were burn-out, depression, distress, suicidal ideation, and substance abuse. A meta-analysis by O'Connor et al. (16) (33 studies, *N* = 9,409) described three dimensions of burn-out: “the prevalence for emotional prostration was 40% [Confidence Interval (CI) 31–48%]”, “for depersonalization was 22% (CI 15–29%), and for reduced personal performance level was 19% (CI 13–25%)”. Mateen and Dorji (14) described a prevalence of burn-out of about at least 25% and associated burnout complaints with workload and relationships at work. Clarity of role, professional autonomy, being treated fairly, and access to regular supervision appear to be protective.

#### Mental health issues amongst HCW as a result of the COVID-19 pandemic and their prevalence during COVID-19

Table 2B displays the total amount of research papers which were reviewed by AvdB, 2nd LvdH, third YT, in order to answer the question “which mental health issues amongst HCW were prevalent as a result of the COVID-19 pandemic during COVID-19?”. This search yielded 345 peer-reviewed journal articles. Four duplicate articles were deleted. Using the inclusion and exclusion criteria, after screening titles and abstracts, 305 papers were excluded that were not relevant to the specific question (e.g., studies related to the wrong population (all people, children), not specified to HCW, population not Anglo-Saxon and therefore difficult to compare with respect to mental health problems, other pandemics like SARS and Ebola, language not English or Dutch). After double blind screening abstracts for eligibility, another 24 articles were excluded [irrelevant topics, like intervention studies or effect studies) (Randomized Controlled Trials)]. In total, 14 English articles were reviewed. All of them described the relation between COVID-19 and mental health problems in HCW and eight of them described the (pooled) prevalence of mental health problems during COVID-19 within HCW as well.

The 345 peer-reviewed journal articles associated with the mental health problems of HCW were published in the period January 1st 2020 till December 1st 2021. From the 14 articles included in this study, 13 of them were systematic reviews and/or meta-analyses and one of them was a field study. These studies confirmed that the COVID-19 pandemic had an impact on the prevalence of mental health problems of HCW. Most frequently used mental health outcomes were depression, insomnia, distress, and anxiety (or related symptoms) (4, 19–23). The prevalence of anxiety or Post Traumatic Stress Disorder (PTSD) was mentioned in 12 studies and the point prevalence ranged from 14 to 67% (fear related symptoms) and was related to COVID-19 exposure and the lack of protective equipment, especially by those on the frontline and nurses in various sectors of healthcare (24, 26–29). Women appeared to be more emotionally affected by the pandemic than men; they reported higher levels of anxiety symptoms and burnout. Depression rates were reported secondary to anxiety phenomena in 10 studies, with prevalence ranging from 24 to 30% (25, 30). Most of the studies described depression as a consequence of ill-treated burnout complaints or underestimated burnout complaints (31). The burnout complaints were associated with the extremely high workload, moral injury, practice changes and financial impacts. Prevalence of distress was mentioned in two studies, ranging from 50 to 56%. Distress was measured by questions about worrying, somatic complaints, and fatigue. The prevalence of poor sleep quality and insomnia (ranged from 41to 44%) was reported in five studies.

#### Sustainable employability of HCW in healthcare

Table 2C displays the total amount of research papers reviewed by YT and LvdH, third AvdB, fourth LdV in order to explore the relationship between mental health and SE of HCW. This search yielded 356 peer-reviewed journal articles. Three articles were added from the expertise network of the authors as described in the methods section. Thereby, two duplicates were deleted. Using inclusion and exclusion criteria, first after screening titles and abstracts, 346 papers were excluded that were relevant to the research question (e.g., studies related to the wrong population, not specified to HCW). After double blind screening abstracts for eligibility, another five articles were excluded [irrelevant topics, like intervention studies or effect studies (RCT's)]. In total, six articles were reviewed. Five of them described SE of HCW in healthcare according to the scientific literature and three of them described the relationship between mental health of HCW and SE in healthcare.

SE of HCW is defined in many different ways, but the literature search did not yield many results within the period of January 1st 2020 till December 1st 2021. As summarized in all the articles, SE is related to keeping the HCW in the healthcare sector so continuity of care can be guaranteed.

Two of the articles had a positive approach to achieve SE, namely that SE is the creation of challenging jobs with adequate management support and steering by HCW themselves on job satisfaction and work ability (33, 36). In three of the articles SE was related to the health of the HCW themselves by doing their job while being mentally and physically healthy.

Three of the articles outlined the relationship between burnout and SE (32, 34, 37). In these three articles the increase in stress explained the cause of burnouts. In the six included articles in the study the increase in workload in the healthcare sector was described as a threat to SE. There was an overall negative relation between (mental) health and SE. Roczniewska and Richter (33) argued that “SE has three equivalent facets: productivity, both mental and physical health, and nice working conditions”. Smyth and Pit (35) stated that a work-life balance affects these facets: a good work-life balance improves psychological and mental health. This improved mental health affects attitude to work and workload, which subsequently leads to a better work ability and SE.

## Discussion

Several studies explored mental health within HCW. In this systematic review, three separate meta-analyses were conducted to explore the central research question. First of all, the distinction between the period before and during the pandemic and the prevalence of mental health problems amongst HCW was made.

It is evident that the impact of the COVID-19 pandemic on the mental health of HCW is huge. Although mental problems in HCW like burn-out, depression, distress, suicidal ideation, and substance abuse were mentioned before the pandemic (8, 10, 12, 16, 17), during the pandemic the levels of mental health symptoms increased. Most eye-catching were the increasing levels of burn-out, fear, and anxiety when compared to the mental health complaints before the COVID-19 pandemic (19, 24, 26, 27). Uncertainty and fear (to bring infection home due to lack of protective equipment and/or intensive interaction with COVID-19 patients) resulted in an increase in distress (31, 37). As the pandemic continued, depression, insomnia, and/or PTSD complaints increased (19–22, 28). The increase of depression, anxiety, and PTSD increases the risks of developing other diseases and suicide (38), which requires government and policy makers to strengthen their mental health systems.

The moderating effects of COVID-19 on the mental health of HCW seemed to be significant. The third meta-analysis confirmed a negative relation between (mental) health and SE. As COVID-19 brought a surplus of patients with complex management needs, the workload mentioned by HCW was high (more intensive treatment and more hours a day over a long period) (32, 33, 35, 36). The adaptations to fit the unpredictable context in healthcare have had a significant impact on HCW. The moderating effects of COVID-19 on mental health found in some studies were related to the changed perspective of HCW on their personal life and loyalty to work. For instance, de Vroege and van den Broek (37) reported that 4.3% of the questioned HCW in their survey were considering quitting their job in healthcare, whilst others were thinking of reorganizing their job or working more from home (56.6 and 31.5%, respectively). The latter finding should be considered positive in light of resilience; the results suggest that HCW are able to reorganize their work to their needs. Nevertheless, considering the present results it can be stated that COVID-19 has a negative moderating effect on mental health and therefore a negative impact on the SE of HCW.

The resilience of an organization is largely determined by the resilience of its workforce. Therefore, hospitals should assume their responsibility to act and protect their workers during pandemics (39). Amidst these many sources of strain, Fleuren et al. (40) recommend attention and understanding for how to protect HCW health and wellbeing given the need to sustain the functioning of HCW. Previously, the strategies to fight COVID-19 were about everyone's general health. It soon became clear, however, that these strategies should also incorporate prevention and treatment of HCW. An earlier study based on another pandemic (41) found that institutional context has strong effects on workforce psychological outcomes. Their results imply that when government and health providers take responsibility for protecting their HCW regarding mental health, this creates a good breeding ground for mutual trust and loyalty to the employer. Furthermore, the results from Maunder et al. (42), based on the Severe Acute Respiratory Syndrome (SARS) pandemic, proposed an approach to alleviate the stress experienced by HCW by organizing psychological support, focused on both organizational and individual characteristics. Shanafelt et al. (43) reported that next to transparent communication and precautionary measures, social support outside the workplace and maintaining social contact reduced the likelihood of emotional distress and work absenteeism. In addition, Fleuren and Poesen (39) stated that an important individual protective factor in HCW mental health is personal resilience. Providing mental healthcare to their colleagues through psychosocial support will increase resilience. Also, training young HCW on how to deal with all sources of strain will create resilience and act in a preventive manner for burnout.

The COVID-19 pandemic has psycho-social, physical, and technical implications for HCW as they need to adapt to a drastically changed work environment (44, 45). It is critical for organizations, in light of COVID-19, to understand how changes affect the experience of HCW and how a potential misfit with organization performance can be resolved through the development of HRM activities (46). In addition, the role of the manager is crucial and it is advisable for organizations to invest in HRM strategies that increase competencies in leadership styles such as transformational-, participatory-, and servant leadership (43, 47). A specific focus on improving mental health can be achieved by providing feedback, regulatory space, and social support in the workplace (48). In addition to empathy, it is also important that managers master other skills such as honest and transparent communication, remaining calm and leading by example, helping HCW stay safe and healthy, and being factual, (49). Finally, Fleuren and Poesen (39) reported that “a team's social climate as a good functioning contextual resource was related to less depressive complaints and appeared to diminish the association between worrying about getting infected and depressive symptoms”.

## Strengths and limitations

Several strengths of this study are worth mentioning. First, to our knowledge, this is the first systematic review that combined mental health issues in HCW before and during the COVID-19 period in relation to SE. Second, a comprehensive search strategy was used to identify relevant studies, which led to analysis of 1,126 studies, of which 32 studies were included to answer the research questions. Finally, the results of this study do have high potential and should have a great impact on future policy.

The limitations of this study were six-fold. First, most included studies had a cross-sectional design which limited the possibility to describe the change over time of mental health status: no causal conclusions can be made based upon these findings. COVID-19 is still a worldwide pandemic, meaning that long-term impact on mental health remains hypothetical. In line with COVID research, you are always behind in advance: the virus, people, and circumstances change, and therefore research structurally runs behind. The COVID-19 pandemic continues and its complete effect on mental health is uncertain. Another limitation of this study is its heterogeneity regarding the included populations, which makes a careful interpretation of the key findings necessary. Possible selection bias may also have occurred since HCW that were not able to use certain platforms. Relevant research regarding SE was scarce and various definitions of sustainability were used. Finally, an important limitation is the fact that different instruments have been used in the description of the various themes.

## Conclusion and recommendations

In conclusion, this systematic review showed that mental problems amongst HCW were already abundantly present before the COVID-19 pandemic. However, mental health problems have increased in prevalence, severity, and variation. The findings showed that mental health problems have moderating effects on the mental health of HCW and therefore on SE: absenteeism has increased and perspective on work has changed. It is time to prioritize mental health in order to prevent acute care capacity from declining even further and ending up in a vicious circle. Therefore, a plea is made for the use of preventive activities and standardized attention for mental health in professional training and working conditions of HCW. Furthermore, it is not yet known which (or if) different profession groups suffer from different mental health complaints compared to other profession groups, but this difference should be explored in the future. As a result, further research should also focus on what kind of training should be developed, because COVID-19 impacted young HCW to a great extent (50, 51).

To prevent HCW from experiencing mental health problems and maintain SE during the pandemic waves, a systematic mental health tool should be used frequently. Screening HCW with regards to mental health problems provides early protection. Moreover, we recommend longitudinal follow-up of mental health problems of HCW in the context of SE. Therefore a validated, standardized mental health scoring tool is needed. A scale for work-related anxiety symptoms and stress, related to the COVID-19 pandemic, has recently been designed by Chung and Kim (52). To realize pandemic preparedness, mental health of HCW is an essential precondition for SE. The COVID-19 pandemic requires decisiveness from managers in making choices regarding HRM activities that are aimed at the wellbeing of the HCW. Attention to increasing psychological wellbeing by expanding energy sources in the workplace is necessary. This pandemic has highlighted the need to raise awareness of the mental health of HCW in order to achieve SE. Therefore, the presence of mental health professionals in policy and government task forces is necessary.

The COVID-19 pandemic challenged the healthcare field and emphasized the need to be better prepared for future pandemics.

## Author contributions

All authors contributed to the study and read and approved the final manuscript.
